# CD4 Cell Levels during Treatment for Tuberculosis (TB) in Ethiopian Adults and Clinical Markers Associated with CD4 Lymphocytopenia

**DOI:** 10.1371/journal.pone.0083270

**Published:** 2013-12-16

**Authors:** Sten Skogmar, Thomas Schön, Taye Tolera Balcha, Zelalem Habtamu Jemal, Gudeta Tibesso, Jonas Björk, Per Björkman

**Affiliations:** 1 Infectious Diseases Research Unit, Department of Clinical Sciences, Faculty of Medicine, Lund University, Malmö, Sweden; 2 Department of Medical Microbiology, Faculty of Health Sciences, Linköping University, Linköping, Sweden; 3 Department of Clinical Microbiology and Infectious Diseases, Kalmar County Hospital, Kalmar, Sweden; 4 Ministry of Health, Addis Ababa, Ethiopia; 5 Oromia Regional Health Bureau, Addis Ababa, Ethiopia; 6 Columbia University Mailman School of Public Health, International Center for AIDS Care and Treatment Programs- Ethiopia, Addis Ababa, Ethiopia; 7 Research and Development Unit, Skåne University Hospital, Lund, Sweden; National Institute for Infectious Diseases (L. Spallanzani), Italy

## Abstract

**Background:**

The clinical correlations and significance of subnormal CD4 levels in HIV-negative patients with TB are unclear. We have determined CD4 cell levels longitudinally during anti-tuberculosis treatment (ATT) in patients, with and without HIV co-infection, and their associations with clinical variables.

**Method:**

Adults diagnosed with TB (maximum duration of ATT for 2 weeks, and with no history of antiretroviral therapy (ART) in HIV-positive subjects) were included consecutively in eight out-patient clinics in Ethiopia. Healthy individuals were recruited for comparison at one of the study health centers. Data on patient characteristics and physical findings were collected by trained nurses following a structured questionnaire at inclusion and on follow-up visits at 2 and 6 months. In parallel, peripheral blood CD4 cell levels were determined. The evolution of CD4 cell levels during ATT was assessed, and the association between clinical characteristics and low CD4 cell levels at baseline was investigated using regression analysis.

**Results:**

In total, 1116 TB patients were included (307 HIV-infected). Among 809 HIV-negative patients, 200 (25%) had subnormal CD4 cell counts (<500 cells/mm^3^), with <350 cells/mm^3^ in 82 (10%) individuals. CD4 cell levels increased significantly during the course of ATT in both HIV+ and HIV- TB-patients, but did not reach the levels in healthy subjects (median 896 cells/mm^3^). Sputum smear status, signs of wasting (low mid upper arm circumference (MUAC)), and bedridden state were significantly associated with low CD4 cell counts.

**Conclusion:**

A high proportion of Ethiopian TB patients have subnormal CD4 cell counts before starting treatment. Low CD4 cell levels are associated with smear positive disease and signs of wasting. The continuous increase of CD4 cell counts during the course of ATT suggest a reversible impact of active TB on CD4 cell homeostasis, which may be considered in interpretation of CD4 cell counts in HIV/TB co-infected subjects.

## Introduction

The majority of persons co-infected with TB and HIV (79% of 1.1 million patients in 2011) live in sub-Saharan Africa [1]. HIV-infected individuals have a high risk of developing active TB following infection, and also have increased mortality. Initiation of ART during the course of ATT has been shown to reduce mortality in co-infected persons, especially in those who are severely immunosuppressed [2]–[4], and is recommended in current WHO guidelines [5].

Absolute CD4 cell levels are the main markers for disease severity in patients with HIV, as well as the best markers yet for disease progression [6]. The time for initiation of ART is based on these levels, also for patients with concomitant TB [5]. The reference range of CD4 cell counts is broad, and these counts can be affected by several factors [7]. Some studies have observed a lower range of CD4 cell counts in apparently healthy subjects in regions of sub-Saharan Africa than the reference range in Caucasian populations [8], [9], suggesting the existence of geographical variations. Furthermore, low CD4 cell counts in HIV-negative patients with TB have been described from different settings, suggesting that TB by itself could have an impact on CD4 cell homeostasis; however, the mechanism, clinical correlations or significance of this phenomenon are not well understood [10]–[12].

We have recently found that low CD4 cell count strata are strongly correlated to signs of wasting among HIV-positive Ethiopian adults with TB (unpublished data). In order to estimate the contribution of TB per se to the associations between clinical parameters and CD4 cell counts, we have prospectively followed these variables in TB-patients, with and without HIV co-infection recruited in Ethiopian health centers.

## Materials and Methods

### Ethics statement

All subjects provided written informed consent prior to inclusion into the study. No interventions interfering with standard care were done during the study period, with the exception of CD4 cell count analysis of HIV-/TB individuals. The study was approved by the National Ethics Review Committee at the Ministry of Science and Technology of Ethiopia and by the Ethical Review Board at Lund University, Sweden.

### Study design and setting

This prospective cohort study was conducted in outpatient TB clinics in the Oromia region, Ethiopia. Adult patients with TB were recruited at six health centers and two hospitals between September 2010 and September 2012. A reference group of healthy HIV-negative subjects were recruited consecutively from a voluntary HIV counseling and testing (VCT) facility in one of the health centers.

### Diagnosis and treatment of patients with TB

Patients were diagnosed with TB according to Ethiopian National Guidelines [13]. Three sputum smears were obtained from each patient. Smear positive pulmonary TB was defined by the detection of acid-fast bacilli (AFB) in at least one out of three sputum smears. The diagnosis of smear negative pulmonary TB required two series of negative sputum smears, non-response to broad-spectrum antibiotics, as well as clinical presentation and chest X-ray compatible with pulmonary TB. For a diagnosis of peripheral lymphnode TB, fine-needle aspirate cytology consistent with the diagnosis was required. Other forms of extrapulmonary TB were diagnosed using targeted investigations, depending on organ manifestation.

ATT was provided as directly observed treatment (DOTS) with daily visits to the clinic during the intensive phase treatment. From September to December 2010, an eight-month course of rifampicin, isoniazid, pyrazinamide and ethambutol was given for two months, followed by isoniazid and ethambutol for 6 months. This regimen was then changed to a standard short-course regimen (two-month treatment with all four drugs with a four-month continuation phase of rifampicin and isoniazid).

### HIV testing

HIV testing is recommended for all patients with confirmed or suspected TB in Ethiopia, and is performed using rapid tests by TB clinic staff through provider-initiated testing. During the study period, KHB tests (Kehua Bio-engineering Co, Shanghai, China) were used for screening. Positive results with this test were confirmed with Statpack (HIV 1/2 Stat-Pak, Chembio Diagnostic Systems, New York, USA); if this test showed a negative result, further testing was done with Unigold (Uni-Gold TM HIV, Trinity Biotech, Wicklow, Ireland). In case of a positive test, patients were referred to an ART clinic in the same facility for enrolment in HIV care and consideration of ART initiation.

### Methods

The following inclusion criteria were applied: age 18 years or older, TB diagnosed according to national guidelines, residence in the clinic catchment area and consent to HIV testing. Exclusion criteria were: having received ATT for more than 2 weeks for the current episode of TB at the time of inclusion, previous ATT within the preceding 6 months, or ART of any duration. TB-clinic nurses, who received detailed and repeated training by the research group members on the study protocol, performed all study investigations.

The study protocol included questions on disease history and symptoms (bedridden state, hospitalization, cough, dyspnea, fever, weight loss, anorexia, lymph node enlargement, skin rash, diarrhea and odynophagia). The physical examinations focused on findings potentially associated with immunosuppression, including conjunctival pallor, oral candidiasis, oral hairy leukoplakia (OHL), gingivitis, cervical lymphadenopathy, skin rash (without further specification) and herpes zoster scar. MUAC and BMI were used as markers for wasting and were collected, using scales and wall mounted measuring sticks for BMI calculation and dedicated measuring bands for measurement of MUAC provided to the health centers.

Follow-up examination was performed following the same procedure after 2 and 6 months of ATT. Treatment outcome was defined according to WHO based national guideline criteria, i.e. treatment completion, cure, death, treatment failure, default and transfer out [5]. The date of ART initiation was noted for those HIV patients who started such treatment during the follow-up period.

Consenting healthy individuals were recruited at a VCT clinic located in one of the study health centers. These subjects were required to be 18 years of age or older, have a negative HIV rapid test and have no known chronic illness or any symptoms suggestive of TB or acute disease. They were recruited and interviewed by a trained peer counselor using a structured questionnaire, with measurement of BMI and MUAC.

Blood samples were obtained for CD4 cell count analysis at baseline, and were repeated at 2 and 6 months for all TB-patients. CD4 cell count flow cytometry was performed at two central laboratories (Adama regional laboratory and Bishoftu hospital laboratory) using FACSCount and FACSCaliber (Becton Dickinson). Regular monitoring and external quality assurance tests of the machines were performed regularly. For the purpose of this study, HIV testing was repeated after 6 months in patients testing negative for HIV at baseline if initial CD4 cell counts were below 350 cells/mm^3^.

### Data collection and statistical analysis

Data was collected on paper forms and was entered into a Microsoft Excel® database and crosschecked before transfer to IBM SPSS® V.20, which was used as a base for all statistical analysis. Baseline characteristics were reported as frequencies, percentages or median values. Wilcoxon signed rank test was used for statistical test for significance between observations at different time points. Two threshold levels were used for definition of low CD4 cell counts in HIV-negative TB patients (HIV-/TB) subnormal CD4 cell counts (below 500 cells/mm^3^; since this is the lower normal reference value), and CD4 lymphocytopenia (below 350 cells/mm^3^;the current recommended threshold for starting ART in HIV+ patients).

A univariate analysis of all variables was performed. For this analysis, BMI and MUAC were categorized according to the median of the HIV-negative patients. Variables with a p-value of less than 0.3 were entered into a multivariable regression analysis, adjusting for age and gender.

For TB patients, we analyzed development of CD4 levels during ATT. For HIV-positive TB patients (HIV+/TB), only those who did not start ART during the follow-up period were included for this analysis to avoid the effect of ART on CD4 cell count evolution. A change of at least 50 cells/mm^3^ between observation time points was used to define increasing or decreasing CD4 cell counts.

## Results

### Baseline characteristics

Out of 2135 patients registered in the clinics during the study period, 1116 (52%) were included, whereas 1019 had at least one exclusion criterion. Among these, 225 (22%) were under 18 years of age, 127 (12%) did not consent to participation, 60 (6%) did not consent to HIV-testing, 94 (9%) resided outside the catchment area, and 309 (30%) had received more than 2 weeks of ATT at the time of screening for eligibility. Among HIV-positive subjects, 284 were on ART at the time of TB diagnosis. Seventeen participants were excluded since they did not provide blood for baseline CD4 cell count. Furthermore, for 81 HIV-/TB patients, follow up CD4 samples at either 2 or 6 months were missing and those patients were only included in the baseline analysis.

Of the 1116 patients included, 307 (28%) were HIV positive and 166 (54%) of these started ART during the 6-month follow up period. Baseline characteristics of the study participants are presented in *table 1*. The HIV positive and negative subjects were comparable in distribution of age, gender, residence and occupational distribution. There was a non-significant increased frequency of smear positive pulmonary TB among HIV-/TB patients, and conversely a higher frequency of smear negative pulmonary disease in HIV+/TB patients. The healthy individuals tended to be younger and have rural residence, but the gender distribution was similar.

**Table 1:** Baseline characteristics of 1116 patients with TB with and without HIV and 298 healthy individuals.

		All TB patients (n = 1116)	HIV+/TB patients (n = 307)	HIV-/TB patients (n = 809)	Healthy individuals (n = 298)
Median Age (years; IQR)		28 (22–28)	32 (28–40)	29 (22 – 42)	23 (20 – 28)
Male gender, n (%)		724 (51.2)	156 (50.8)	432 (53.4)	136 (54.4)
Occupation	Sales and services	96 (8.6)	35 (11.4)	61 (7.5)	19 (6.6)
	Manual labor	335 (30)	113 (36.8)	222 (27.4)	216 (74.5)
	Agriculture	208 (18.6)	43 (14.0)	165 (20.4)	53 (18.3)
	Unemployed	477 (42.7)	116 (37.8)	361 (44.6)	2 (0.7)
Residence, n (%)	Urban	943 (84.7)	274 (89.5)	669 (82.9)	182 (65.5)
	Rural	170 (15.3)	32 (10.4)	138 (17.1)	96 (34.5)
Type of TB, n (%)*	Smear positive	405 (36.3)	99 (32.2)	306 (37.8)	-
	Smear negative	310 (27.8)	94 (30.6)	216 (26.7)	-
	Lymph node TB	296 (26.5)	87 (28.3)	209 (25.8)	-
	Other location of TB	125 (11.2)	36 (11.7)	89 (11.0)	-
Previous TB		47 (3.3)	7 (2.3)	40 (4.9)	-
Median CD4 cell count (IQR)		639 (376 – 890.3)	173 (95 – 336)	671 (500 – 883.5)	897 (700 – 1083)
Median CD4 percentage (IQR)		35 (25–41)	12 (8 – 18)	37 (30.9 – 43.0)	37 (33 – 43)

*20 patients had both a diagnosis of pulmonary and extrapulmonary TB.

Among HIV+/TB patients the median CD4 cell count was 173 cells/mm^3^ (IQR 95-336). The median CD4 cell count was lower in the HIV-/TB patients compared to reference subjects, 671 cells/mm^3^ (IQR 500–883.5) vs. 896 cells/mm^3^ (IQR 700–1083). Two-hundred HIV-/TB patients (25%) had CD4 cell counts below 500 cells/mm^3^, and 82 (10%) had CD4 cell counts lower than 350 cells/mm^3^.

### Evolution of CD4 cell counts during ATT

**Figure 1:**
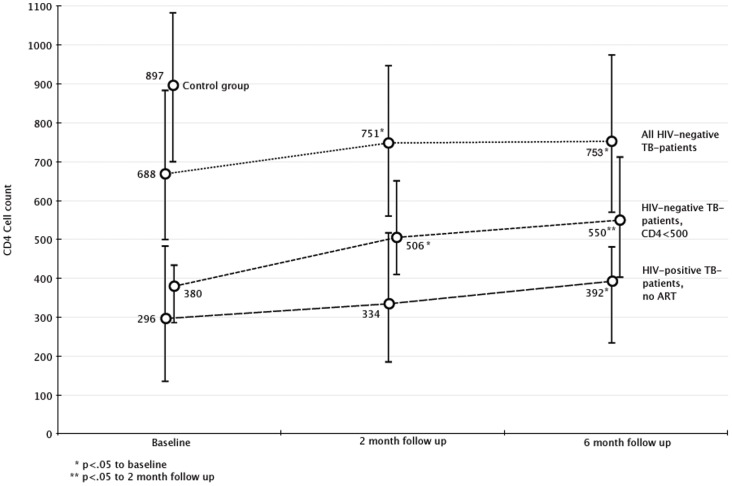
Evolution of CD4 cell counts in HIV negative and positive patients with TB during treatment for TB. The figure shows median CD4 levels and the bars represent IQR. Only patients with follow up CD4 are included in this analysis, and for the control group only baseline CD4 was measured. For HIV+ patients, only patients who did not start ART and had follow up CD4 were included (n = 71).

Among 472 HIV-/TB patients with follow-up results for CD4 cell counts during ATT, the median counts increased from 688 cells/mm^3^ (IQR 497–917) to 753 cells/mm^3^ (*figure 1*). Patients without follow-up CD4 cell count results (n = 337) had similar median baseline CD4 cell counts (666 cells/mm^3^; IQR 508–859).

The rise in CD4 cell counts was even more pronounced in HIV-/TB patients with low baseline CD4 cell counts; in HIV-/TB patients with CD4 cell counts less than 500 cells/mm^3^ these counts increased from a median of 380 to 550 cells/mm^3^ after 6 months. In contrast to the other subgroups (*figure 1*), these patients also manifested a significant increase throughout treatment (both between baseline and 2 month follow up as well as between 2 and 6 month follow up). For HIV+/TB patients the increase was significant from baseline to either 2 month or 6-month follow up.

Although the majority of HIV-/TB patients with CD4 cell counts below 500 cells/mm^3^ had increasing CD4 cell levels during treatment (n = 87), 24 of these subjects did not show this pattern, and in 8 patients these levels even decreased. The characteristics of these subgroups of HIV-/TB patients are presented in *table 2* *and* *3*. No clear differences in the distribution of such characteristics were observed between these subgroups.

**Table 2:** Characteristics of HIV-/TB patients and CD4 cell count of less than 500, divided by either increase, decrease or stagnant CD4 cell count after 6 months ATT.

	Increase after 6 month (n = 87)			Decrease after 6 month (n = 8)			No change after 6 month (n = 24)		
	Baseline	2 Month	6 month	Baseline	2 Month	6 month	Baseline	2 Month	6 month
Age	29			36			24		
Male gender, n (%)	54 (62.1)			7 (87.5)			14 (58.3)		
Smear positive TB, n (%)	39 (44.8)			3 (37.5)			9 (37.5)		
Smear negative TB, n (%)	15 (17.2)			3 (37.5)			6 (25)		
Lymph node TB, n (%)	23 (26.4)			0			6 (25)		
Other location of TB, n (%)	10 (11.5)			2 (25)			3 (12.5)		
Median BMI kg/height^2^	18.4	19.1	19.8	17.7	19.0	18.8	17.2	18.4	18.7
Median MUAC (cm)	21.5	22.0	23.0	21.0	21.8	22.0	20.0	22.0	22.5
Median CD4 cells/mm^3^	372	557	605	424.5	448	342.5	400	435	406
Median Percentage	29.6	-	32.4	32.0	-	37.0	33.5	-	30.5

*Increase or decrease defined as >50 cells/mm^3^. Only patients who had follow up CD4 cell counts were included in the analysis

**Table 3:** Characteristics of HIV+/TB patients not starting ART divided by either increase, decrease or stagnant CD4 cell count after 6 months ATT.

	Increase after 6 month (n = 35)			Decrease after 6 month (n = 19)			No change after 6 month (n = 17)		
	Baseline	2 month	6 month	Baseline	2 month	6 month	Baseline	2 month	6 month
Age	35 (28–38)			31			32		
Male gender, n (%)	19 (54.3)			8 (42.1)			8 (47.1)		
Smear positive pulmonary TB, n (%)	9 (25.7)			7 (36.8)			4 (23.5)		
Smear negative pulmonary TB, n (%)	13 (37.1)			7 (36.8)			5 (29.4)		
Lymph node TB, n (%)	5 (14.3)			6 (31.6)			6 (35.3)		
Other location of TB, n (%)	5 (14.3)			2 (10.5)			2 (11.8)		
Median BMI kg/height2	17.8	18.5	19.6	18.5	19.7	19.6	18.4	18.8	19.9
Median MUAC (cm)	20	21	22.5	22	23	23	21	22	24
Median CD4 cells/mm^3^	188	288	417	505	505	407	243	235	249
Median Percentage	11.0		15.0	23.0		18.0	13.0		12.0

Increase or decrease defined as >50 cells/mm^3^. Only patients who had follow up CD4 cell counts were included in the analysis

For HIV+/TB patients who did not start ART (n = 71), overall median CD4 cell counts increased, but for these subjects the pattern was more heterogeneous than that found in HIV-/TB patients. In 35 cases CD4 cell counts had increased after 6 months, while these counts had decreased for 17 patients, and remained at similar levels for 19 patients. HIV+/TB patients with increasing CD4 cell counts had lower baseline levels (188 cells/mm^3^) than those with decreasing CD4 cell counts (505 cells/mm^3^).

### Clinical characteristics of HIV-/TB patients with low CD4 cell counts

The correlation between clinical parameters and low CD4 cell counts at baseline (less than 500 cells/mm^3^ or less than 350 cells/mm^3^) is presented in detail in *table 4*. Patients with baseline CD4 cell counts below 500 cells/mm^3^ were significantly more likely to have smear positive pulmonary TB (adjusted OR 1.6, IQR 1.2-2-3). Reversely, the prevalence of smear negative pulmonary TB was lower in this group (adjusted OR 0.6, IQR 0.4–0.9). A similar, although not statistically significant, relationship was observed for patients with CD4 cell counts below 350 cells/mm^3^.

**Table 4:** Correlation of clinical parameters to CD4 cell cut off levels in HIV-/TB patients.

		CD4 cell count <500 cells/mm^3^, n = 200			CD4 cell count <350 cells/mm^3^, n = 82		
	Frequency all HIV-/TB patients	Frequency	OR unadjusted	OR adjusted*	Frequency	OR unadjusted	OR adjusted*
Previous TB	40	3	0.2 (0.1–0.8)	0.2 (0.1–0.7)	2	0.5 (0.1–1.9)	0.3 (0.1–1.9)
Smear positive PTB	306	94	1.7 (1.2–2.3)	1.6 (1.2–2.3)	36	1.3 (0.8–2.1)	1.3 (0.8–2.0)
Smear negative PTB	216	41	0.6 (0.4–0.9)	0.6 (0.4–0.9)	20	0.9 (0.5–1.5)	0.8 (0.5–1.4)
Lymphnode TB	209	42	0.7 (0.5–1.0)	0.8 (0.5–1.2)	18	0.8 (0.5–1.4)	0.9 (0.5–1.5)
Cervical lymph node enlargement	120	18	0.5 (0.3–0.8)	0.6 (0.3–1.0)	10	0.8 (0.4–1.5)	0.9 (0.5–1.8)
Cough	509	142	1.6 (1.1–2.3)	1.4 (1.0–2.0)	58	1.5 (0.9–2.4)	1.3 (0.8–2.2)
Bloodstained sputum	162	49	1.4 (1.0–2.1)	1.3 (0.9–1.9)	24	1.8 (1.1–2.9)	1.6 (1.0–2.8)
Shortness of breath	342	92	1.2 (0.9–1.7)	1.2 (0.9–1.6)	38	1.2 (0.7–1.9)	1.1 (0.7–1.8)
Fever	618	159	1.3 (0.9–1.9)	1.3 (0.9–1.9)	67	1.4 (0.8–2.6)	1.4 (0.8–2.6)
Night sweats	627	161	1.2 (0.8–1.9)	1.2 (0.8–1.8)	68	1.4 (0.8–2.6)	1.4 (0.8–2.5)
Conjunctival pallor	93	27	1.3 (0.8–2.1)	1.1 (0.7–1.9)	13	1.5 (0.8–2.9)	1.4 (0.7–2.7)
Diarrhea	34	11	1.5 (0.7–3.1)	1.6 (0.7–3.3)	3	0.9 (0.3–2.8)	0.9 (0.3–2.9)
Diarrhea, recurrent	29	13	2.6 (1.2–5.5)	2.3 (1.1–5.0)	6	2.4 (1.0–6.1)	2.2 (0.9–5.6)
Loss of appetite	558	143	1.2 (0.8–1.7)	1.2 (0.8–1.7)	62	1.4 (0.9–2.4)	1.4 (0.9–2.5)
Odynophagia	102	27	1.1 (0.7–1.8)	1.1 (0.7–1.8)	15	1.6 (0.9–3.0)	1.6 (0.9–3.0)
Significant weight loss	554	144	1.3 (0.9–1.8)	1.2 (0.8–1.7)	65	1.8 (1.0–3.2)	1.7 (1.0–3.1)
Lower than median BMI (<18.5 kg/m^2^)	388	105	1.3 (0.9–1.8)	1.2 (0.8–1.6)	41	1.1 (0.7–1.7)	1.0 (0.6–1.6)
Lower than median MUAC (<22 cm)	400	125	2.0 (1.5–2.8)	1.9 (1.4–2.7)	55	2.3 (1.4–3.7)	2.2 (1.3–3.6)
Hospitalized	15	5	1.5 (0.5–4.5)	1.7 (0.6–5.1)	3	2.3 (0.6–8.3)	2.5 (0.7–9.3)
Bedridden state	102	41	2.3 (1.5–3.6)	2.1 (1.4–3.3)	19	2.3 (1.3–4.1)	2.3 (1.3–4.0)

*Adjusted for age and gender.Only parameters with significance of less than 0.3 in univariate analysis are present in the table. Variables that did not reach this level of significance were: other location of TB, gingivitis, herpes zoster, oral candidiasis, oral hairy leukoplakia and skin rash.

MUAC, being a surrogate marker for wasting, was significantly associated with low CD4 cell counts in multivariate analysis, with a more than twofold chance of having a MUAC below 22 cm with baseline CD4 cell counts below 350 cells/mm^3^ (adjusted OR 2.2, IQR 1.3–3.6). There was a trend between lower BMI and decreasing CD4 cell counts, although this did not reach statistical significance. Furthermore, patients with a history of bedridden state during their current illness were more likely to have low CD4 cell counts (OR 2.3, IQR 1.3–4.0).

### Outcome of ATT

Thirteen deaths were observed among HIV-/TB patients. These subjects did not show significant differences in clinical features or CD4 cell counts as compared to other patients; their median BMI and MUAC values were slightly lower (18.1 kg/m^2^ and 21 cm, respectively) as well as their CD4 cell counts (559 cells/mm^3^).

## Discussion

In this cohort of Ethiopian adults with TB the prevalence of low CD4 cell counts among HIV-negative persons before initiation of ATT was substantial; 25% had CD4 cell counts below 500 cells/mm^3^ and 10% had CD4 cell counts lower than 350 cells/mm^3^, which is the currently recommended lower threshold level for starting ART in HIV-positive subjects. By correlating CD4 cell strata with clinical variables, we could show that low CD4 cell counts are associated with TB disease severity, such as sputum smear positivity, lower MUAC and bedridden state.

Low CD4 cell levels in HIV-/TB patients have been reported previously; in a study on hospitalized TB patients from Senegal, Kony et. al. found CD4 cell counts below 300 cells/mm^3^ in 14% [11]. Fifty-three (7%) HIV-/TB cases from our cohort had CD4 cell levels below this threshold level. In line with our findings on associations with clinical severity reported here, the lower prevalence of CD4 lymphocytopenia in our population could be related to more advanced disease in hospitalized subjects. In contrast to previous findings from factory workers in Wonji, a district in the vicinity of our study area in Ethiopia [9], we did not find subnormal CD4 cell counts among healthy individuals.

The continuous increase of CD4 cell counts during treatment for TB strongly suggests that TB per se contributes to subnormal CD4 cell levels in peripheral blood. Similar findings have been reported by other researchers in HIV-/TB patients both in the African setting and in other geographical locations [11], [14]–[17]; however, the reasons for this phenomenon, or its clinical significance, are not well understood.

In parallel with increasing median CD4 cell counts during ATT, we observed continuous improvements in signs of wasting (both BMI and MUAC), which implies that CD4 cell depletion and wasting are related to similar underlying factors in TB. Associations between signs of wasting and CD4 cell levels have been explored previously, with discrepant results. In Argentinian HIV-/TB patients, Pilheu et al found lower CD4 cell count in subjects reporting significant weight loss (>20%) than in those with better general condition [17]. In contrast, other researchers failed to show an association between BMI and CD4 cell counts in HIV-/TB patients (14, 16). In a study on CD4 cell levels among healthy Ethiopian factory workers, Abuye et al reported lower BMI in subjects with subnormal CD4 cell counts [8].

For the investigation of correlations between CD4 cell counts and clinical variables we used both BMI and MUAC as markers for wasting and malnutrition. In multiple regression analysis, MUAC <22 cm was correlated with low CD4 cell counts (both below 500 and 350 cells/mm^3^) in HIV-/TB patients. BMI did not turn out to be significant, although there was a trend towards lower CD4 cell count with decreasing BMI. This suggests that MUAC may be a more suitable marker for wasting in this patient group than BMI. In a study from Guinea Bissau MUAC showed a stronger correlation to mortality in HIV+/TB patients than BMI [18].

Several studies have shown a relationship between malnutrition and mortality in TB patients [19]–[21]. We did not, however, find associations between ATT outcome and low baseline CD4 cell counts in our HIV-/TB patients, probably due to the low frequency of adverse outcomes. There was also no clear association between signs of wasting and poor outcome.

Interestingly, we have previously found relationships between CD4 cell strata and wasting (particularly MUAC) in HIV+/TB patients from this cohort (unpublished data), a finding that might be useful to identify subjects with severe immune suppression in settings without access to CD4 cell testing. The fact that similar associations between CD4 cell counts and wasting exist in HIV-/TB suggests that TB may contribute to the wasting syndrome commonly found in co-infected patients, as well as contributing to CD4 cell depletion. Our finding that CD4 cell counts increased during ATT in HIV+/TB patients not initiating ART further supports this, though the mechanism of the interaction between wasting and low CD4 cell count remains a matter of debate [22], [23].

In contrast to HIV+/TB, HIV-/TB patients with low CD4 cell counts had normal CD4 cell percentage, suggesting that the observed decreases in absolute CD4 cell counts are related to peripheral blood lymphocytopenia. Possible explanations for this could be pooling of T-cells at the site of infection [24], [25], a direct cytokine mediated suppressive effect on the production of peripheral lymphocytes [26], or effects related to hypermetabolism and malnutrition secondary to TB infection [23], [27].

The recovery rate of CD4 cell counts in HIV-/TB patients with subnormal baseline levels was slow and continuous, and did not reach the levels found in healthy individuals from the same geographical area even at the completion of ATT. This is in agreement with findings from HIV+/TB patients, showing an impact of TB on CD4 cell counts for several years after completion of ATT [28].

In a study from Tanzania, an increase was demonstrated between baseline and 2 months. Between baseline and 5 month follow up CD4 was unchanged for those not receiving ART [14]. Increasing CD4 cell levels were also observed in HIV+/TB patients not starting ART in our cohort. When divided into subgroups of those with increasing and decreasing CD4 cell count, the result was heterogeneous. This may be expected since HIV in itself lowers CD4 cell counts, thus cancelling a potential effect of ATT on CD4 cell count in some patients. The number of HIV+/TB patients not starting ART during ATT was low (n = 71), which limits the interpretation of these findings. However, our data suggest that severe TB (as measured by the degree of wasting) can contribute to CD4 lymphocytopenia in co-infected subjects, and that it may be partly reversed by ATT.

Our study design has allowed us to correlate clinical variables with CD4 cell counts longtitudinally during ATT in patients with TB, both with and without HIV co-infection. This study also has some limitations. Firstly, TB diagnosis was based on Ethiopian guidelines, and did not include microbiological confirmation other than smear microscopy. Consequently, some subjects with smear-negative pulmonary TB and extrapulmonary disease may have had diagnoses other than TB. We were unable to estimate this proportion; however, in a previous study, 78% of patients diagnosed with lymph node TB according to the Ethiopian Guidelines were found to have positive TB cultures from lymph node aspirates [29], suggesting that the fraction of participants with diagnoses other than TB was small. Furthermore, the methods used for TB diagnosis among our patients reflect the actual situation in most resource-limited settings. We cannot exclude the existence of other factors not screened for in this study that may have had an impact on CD4 cell counts. Finally, HIV diagnosis relied on rapid tests (evaluated by WHO in 2004 [30]), which could potentially have produced false negative results from subjects in HIV seroconversion phase. For this purpose, we repeated HIV testing in patients with CD4 cell counts below 350 cells/mm^3^ at the end of ATT. No HIV seroconversions were detected.

In conclusion, we found a high proportion of CD4 cell lymphocytopenia in Ethiopian HIV-negative adults diagnosed with TB. These levels increased during ATT, both in HIV-negative patients and in HIV+/TB patients not initiating ART, indicating an impact of TB on CD4 cell homeostasis. Decreased CD4 cell counts was associated with clinical markers of advanced TB disease, such as sputum smear positivity, low MUAC and bedridden state. These findings suggest that similar factors cause wasting and CD4 cell depletion in peripheral blood in TB.
